# Enhancing the Performance of Quantum Dot Light-Emitting Diodes Using Solution-Processable Highly Conductive Spinel Structure CuCo_2_O_4_ Hole Injection Layer

**DOI:** 10.3390/ma16030972

**Published:** 2023-01-20

**Authors:** Min Ho Park, Min Gye Kim, Jin Hyun Ma, Jun Hyung Jeong, Hyoun Ji Ha, Wonsik Kim, Soohyung Park, Seong Jun Kang

**Affiliations:** 1 Department of Advanced Materials Engineering for Information and Electronics, Kyung Hee University, Yongin 17104, Republic of Korea; 2 Integrated Education Program for Frontier Materials (BK21 Four), Kyung Hee University, Yongin 17104, Republic of Korea; 3 Advanced Analysis Center, Korea Institute of Science and Technology, 5 Hwarang-ro 14-gil, Seongbuk-gu, Seoul 02792, Republic of Korea; 4 Division of Nano & Information Technology, KIST School, University of Science and Technology (UST), Seoul 02792, Republic of Korea

**Keywords:** colloidal quantum dot (QD), light-emitting diode (LED), hole injection layer, spinel, CuCo_2_O_4_

## Abstract

Charge imbalance in quantum-dot light-emitting diodes (QLEDs) causes emission degradation. Therefore, many studies focused on improving hole injection into the QLEDs-emitting layer owing to lower hole conductivity compared to electron conductivity. Herein, CuCo_2_O_4_ has a relatively higher hole conductivity than other binary oxides and can induce an improved charge balance. As the annealing temperature decreases, the valence band maximum (VBM) of CuCo_2_O_4_ shifts away from the Fermi energy level (E_F_), resulting in an enhanced hole injection through better energy level alignment with hole transport layer. The maximum luminance and current efficiency of the CuCo_2_O_4_ hole injection layer (HIL) of the QLED were measured as 93,607 cd/m^2^ and 11.14 cd/A, respectively, resulting in a 656% improvement in luminous performance of QLEDs compared to conventional metal oxide HIL-based QLEDs. These results demonstrate that the electrical properties of CuCo_2_O_4_ can be improved by adjusting the annealing temperature, suggesting that solution-processed spinel can be applied in various optoelectronic devices.

## 1. Introduction

Quantum dots (QDs) have a size-dependent bandgap because of the quantum confinement effect; thus, the emission wavelength of QDs can be easily controlled using this property [1]. Inorganic QDs have narrower electroluminescence (EL) spectra and higher quantum yield (QY) than conventional organic light-emitting materials [2,3]. In addition, QDs have the advantage of low-cost mass production of quantum-dot light-emitting diodes (QLEDs) through solution-based manufacturing technology [4]. Owing to these advantages, QDs are in the spotlight as light-emitting layers for next-generation displays.

To increase the emission performance of QLED, hole and electron injection/transport layers are inserted between the electrodes and the QD emitting layer (EML) [5]. Various studies are being conducted to employ transition metal oxides (TMOs) as the charge transport layer of QLEDs because TMOs have strong moisture and heat resistance. In particular, these studies applied *p*-type TMOs, such as CuO and NiO, as hole injection layers (HIL) or hole transport layers (HTL) [6,7,8], and *n*-type TMOs, such as ZnO and ZnMgO, as electron transport layers (ETL) [9,10] in optoelectronic devices.

In *n*-type TMOs, sufficient majority carrier electrons are generated owing to oxygen defects, and the conduction band maximum (CBM), which is the electron transport path, consists of metal s-orbitals [11]. Contrastingly, in *p*-type TMOs, majority carrier holes are generated in cation vacancies, which require high formation energy [12]. Moreover, the valence band maximum (VBM), which is the transport path of holes, mostly comprises a localized oxygen 2p orbital; thus, the hole conductivity is poorer than that of electrons in the amorphous structure [13,14]. For this reason, when solution-processed TMOs are used as a charge transport layer, it causes charge imbalance to decrease the luminous efficiency of the QLED [15,16]; therefore, studies on TMOs with high hole injection capability are necessary.

Some studies reported that doping with *p*-type oxides increases hole injection [7,17,18]. Cao et al. improved the luminance of a QLED by up to 61,060 cd/m^2^ by doping Cu into a NiO hole injection layer [7]. However, hole injection through doping is limited because excessive doping can degrade the hole conductivity of *p*-type oxides [7,17,18]. Therefore, a new approach is required to increase the hole conductivity of *p*-type TMOs, such as ternary oxides or highly conductive crystal structures, for hole injection.

The spinel structure has the general formula AB_2_O_4_, where A is a divalent metal ion (Cu^2+^, Ni^2+^, Zn^2+^) in the tetrahedral sites and B is a trivalent metal ion (Co^3+^, Al^3+^, Fe^3+^) in the octahedral sites (Figure S1) [19,20,21]. Ternary oxides with spinel structures can be alternatives to many oxides because their optical and electrical properties can be tuned by changing the combination of the metal ions [22]. Spinel oxides have the relatively low activation energy for electron transfer between different cations [23,24]. Thus, spinel oxides such as CuCo_2_O_4_ and NiCo_2_O_4_ have a conductivity of several tens of S/m, and these electrical conductivity values of spinel oxides are much higher than that of binary oxides [25,26]. In the field of photovoltaics, many studies have been conducted using spinel oxides with superb conductivity as charge transport layers [14,19,27]. However, research on spinel oxides is relatively unusual in the QLED field; therefore, it is especially notable that this study improved hole injection using spinel ternary oxides.

In this study, we fabricated highly conductive *p*-type CuCo_2_O_4_ via a solution process using the spin-coating technique and applied it as a HIL to enhance the charge balance of QLEDs. Substantial variations in the oxygen defects were observed in the solution-processed CuCo_2_O_4_ thin films depending on the annealing temperature. The oxygen defect affected the hole concentration; as a result, the VBM of CuCo_2_O_4_ shifted away from E_F_ as the annealing temperature decreased. When CuCo_2_O_4_ was annealed at 200 °C, the QLED performance was improved, and the hole injection was enhanced through better energy-level alignment. To investigate the reason for the difference in CuCo_2_O_4_ electrical characterization according to the annealing temperature, Hall measurements, X-ray photoelectron spectroscopy (XPS), and ultraviolet photoelectron spectroscopy (UPS) measurements were conducted. In addition to confirming the hole-injection ability of CuCo_2_O_4_, a hole-only device (HOD) was manufactured and measured. These results demonstrate that the electrical properties of CuCo_2_O_4_ can be adjusted by modifying the annealing temperature, suggesting that solution-processed spinel can be applied in various optoelectronic devices.

## 2. Experimental Section

### 2.1. CuCo_2_O_4_ Solution Synthesis

First, 0.48 mmol of copper acetate tetrahydrate (Cu(CH_3_CO_2_)_2_·H_2_O, Sigma Aldrich) and 0.96 mmol of cobalt acetate tetrahydrate (Co(CH_3_CO_2_)_2_·4H_2_O, DAE JUNG) were dissolved in 12 mL ethylene glycol monomethyl ether (DAE JUNG). The solution was then sonicated for 10 min at room temperature to completely dissolve the copper and cobalt precursors. Subsequently, 60 μL of ethanolamine (Sigma Aldrich, St. Louis, MI, USA) was added and stabilized by hydrogen bonding using a stabilizer and stirred for 24 h at room temperature. After stirring for 24 h, the 0.04 M CuCo_2_O_4_ solution changed from blue to dark gray (or dark navy).

### 2.2. Device Fabrication

Patterned indium tin oxide (ITO) glass substrates were cleaned via ultrasonication in DI water, acetone, and isopropyl alcohol for 15 min in sequence. The cleaned ITO substrates were treated with ultraviolet-ozone for 20 min to improve the surface hydrophilicity and increase the ITO work function through carbon contamination removal. The CuCo_2_O_4_ HIL solution was spin-coated at 3000 rpm for 60 s. Subsequently, the solvent was dried on a hot plate at 150 °C for 10 min and then annealed for 60 min (200 °C/300 °C) in an ambient atmosphere. As shown in Equations (1) and (2), acetate residues are removed in the pre-annealing and CuCo_2_O_4_ is formed through post-annealing.

(1) $$\mathrm{Cu}{\left({\mathrm{CH}}_{3}{\mathrm{CO}}_{2}\right)}_{2}+2\mathrm{Co}{\left({\mathrm{CH}}_{3}{\mathrm{CO}}_{2}\right)}_{2}+6H_{2}O\;\to \mathrm{Cu}{\left(\mathrm{OH}\right)}_{2}+2\mathrm{Co}{\left(\mathrm{OH}\right)}_{2}+6\;\mathrm{Acetic}\;\mathrm{acid}$$

(2) $$\mathrm{Cu}{\left(\mathrm{OH}\right)}_{2}+2\mathrm{Co}{\left(\mathrm{OH}\right)}_{2}+\frac{1}{2}O_{2}\;\to {\mathrm{CuCo}}_{2}O_{4}$$

A poly[(9,9-dioctylfluorenyl-2,7-diyl)-co-(4,4′-(N-(4-sec-butylphenyl)diphenylamine)] (TFB) dissolved in p-xylene at 1 wt% was spin-coated at 3000 rpm for 30 s onto the CuCo_2_O_4_ HIL, followed by annealing at 180 °C for 40 min. Then, CdSe/ZnS Green QDs (UNIAM, 20 mg/mL) dispersed in toluene were spin-coated at 2000 rpm for 30 s and then annealed at 90 °C for 10 min. The optical bandgap and photoluminescence spectra of the green QDs used to fabricate the QLEDs are shown in Figure S2. ZnO (Avantama, N-10) dispersed in isopropyl alcohol was spin-coated at 2000 rpm for 60 s and then annealed at 90 °C for 10 min. Finally, a 130 nm thick aluminum was deposited by a thermal evaporation at a deposition rate of 3 Å/s under high vacuum through a shadow mask.

A HOD and an electron-only device (EOD) to evaluate the charge injection ability were produced through spin coating-based solution process identical to the QLED manufacturing process. First, pre-cleaned glass/ITO substrate were treated with ultraviolet-ozone for 20 min. The CuCo_2_O_4_ HIL solution was spin coated at 3000 rpm for 60 s. Subsequently, pre-annealing is performed on the hot plate at 150 °C for 10 min and post-annealing is performed annealed for 60 min at 200 °C (or 300 °C) in an ambient atmosphere. Next, TFB HTL solution was spin-coated at 3000 rpm for 30 s onto the CuCo_2_O_4_ HIL, followed by annealing at 180 °C for 40 min. Finally, a 130 nm thick aluminum was deposited by a thermal evaporation at a deposition rate of 3 Å/s under high vacuum through a shadow mask. First, pre-cleaned glass/ITO substrate were treated with ultraviolet-ozone for 20 min. ZnO ETL was spin-coated at 2000 rpm for 60 s and then annealed at 90 °C for 10 min. Then, a 130 nm thick aluminum was deposited by a thermal evaporation at a deposition rate of 3 Å/s under high vacuum through a shadow mask.

### 2.3. Characterization

The electrical properties such as resistivity, carrier mobility, and carrier concentration of the oxide thin film were measured using Hall measurements (HL 5500PC). XPS and UPS spectra were measured using a surface analysis system (Thermo fisher, NEXSA, Waltham, MA, USA) with an Al Kα (1486.6 eV) source for XPS and a He I (21.22 eV) source for UPS. The energy references of the XPS and UPS spectra were calibrated with respect to the E_F_ of clean Au sample. The morphology of the films was characterized using atomic force microscopy (AFM) (S.I.S-GmbH, Berlin, Germany) in the non-contact mode. The transmittance of the thin films was measured using a UV-visible spectrometer (Cary 100, Agilent). The electroluminescence properties of the QLEDs were measured using an I-V-L system (M-6100, McScience) installed with a source meter (Keithley 2400) and spectroradiometer (CS-2000, Konica Minolta). QLED cross-section images were obtained using HR-TEM, and the elemental composition of the cross-sections was analyzed using energy-dispersive spectroscopy (EDS) data using a field emission electron microscope (JEM-2100F, JEOL).

## 3. Results and Discussion

### 3.1. Characterization of CuCo_2_O_4_ Thin Films

Table 1 shows the dependence of the electrical properties of CuCo_2_O_4_ on the annealing temperature, as measured by Hall measurements using van der Pauw method. The resistivity, mobility, and hole concentration of CuCo_2_O_4_ annealed at 200 °C (CuCo_2_O_4_-200) were 2.295 Ω·cm, 0.96 cm^2^/V·s and 2.834 × 10^18^ cm^−3^, respectively. Then, the resistivity, mobility, and hole concentration of CuCo_2_O_4_ annealed at 300 °C (CuCo_2_O_4_-300) were 0.3917 Ω·cm, 0.72 cm^2^/V·s, and 2.214 × 10^19^ cm^−3^, respectively. The resistance of the binary oxide CuO was too high, making Hall measurements impossible. It was confirmed that both CuCo_2_O_4_-200 and CuCo_2_O_4_-300 films have enough higher conductivities than binary oxides. The relationship between the resistivity (*ρ*), conductivity (*σ*), mobility (*μ*), carrier concentration (*n*), and electrical charge (*q*) of the carriers in metal oxides is shown in Equation (3) [28]:

(3) $$\frac{1}{\rho }=\sigma =nq\mu$$

The conductivities of CuCo_2_O_4_-200 and CuCo_2_O_4_-300, calculated using Equation (1), were 0.4357 S/cm and 2.553 S/cm, respectively. The difference in electrical conductivity of CuCo_2_O_4_-200 and 300 were predominantly caused by the hole concentration because the difference in mobility between the two thin films is not significant. Hall measurement results show that both CuCo_2_O_4_-200 and CuCo_2_O_4_-300 films have relatively enough high electrical conductivity compared to conventional oxides; therefore, the CuCo_2_O_4_ HIL is expected to greatly contribute to hole injection. However, the hole concentration of CuCo_2_O_4_ thin films vary according to the annealing temperature. Therefore, it is expected that the QLED performance depends on the annealing temperature of the CuCo_2_O_4_ HIL owing to the difference in energy level alignment of CuCo_2_O_4_ HILs.

**Figure 1:**
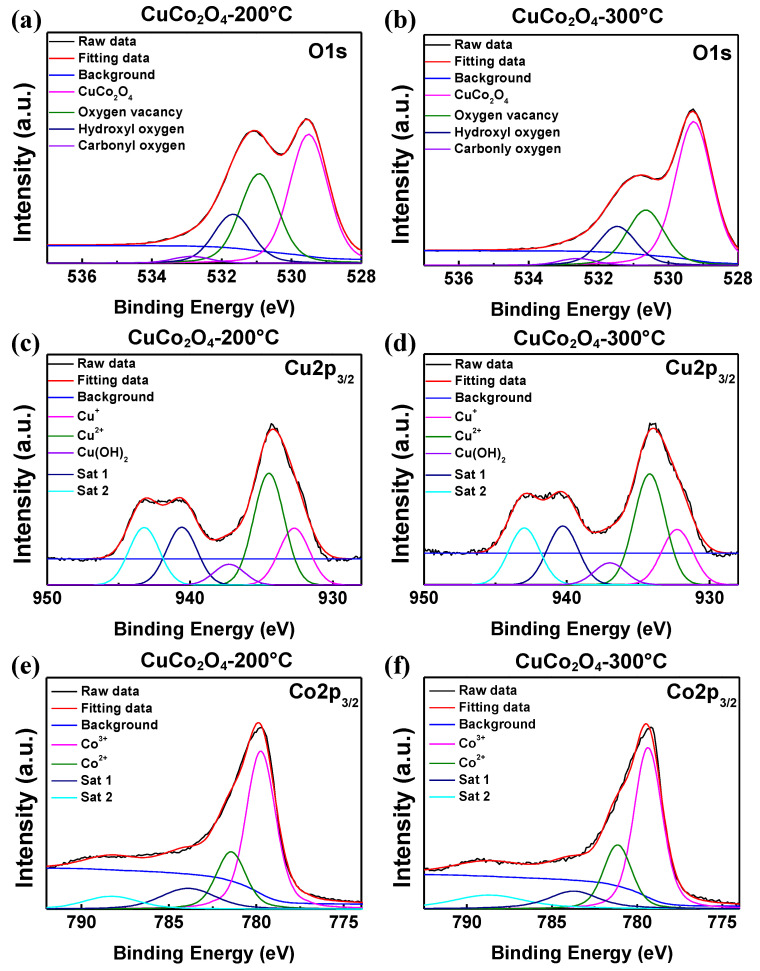
(**a**,**b**) O 1s (**c**,**d**) Cu 2p3/2 and (**e**,**f**) Co 2p3/2 XPS spectra of solution-processed CuCo_2_O_4_ with different annealing temperature.

Figure 1 displays the O 1s, Cu 2p_3/2_ and Co 2p_3/2_ core-level XPS spectra of ITO/CuCo_2_O_4_ at different annealing temperatures of 200 °C and 300 °C. The binding energies of the XPS spectra were calibrated using the C 1s peak of adventitious carbon (284.6 eV as a reference). The O 1s core level was separated into oxide peaks, oxygen vacancies, surface hydroxyl oxygen, and carbonyl oxygen. As seen in Figure 1a,b, more oxygen vacancies and hydroxyl oxygen peaks were observed in the CuCo_2_O_4_-200 specimen than in the CuCo_2_O_4_-300 specimen, which means that more oxygen defects occurred. The tendency of oxygen defects in the CuCo_2_O_4_ to increase at the lower annealing temperature is similar to reported solution-processed metal oxides [29,30]. Cu^+^ ions do not cause satellite peaks because the 3d orbitals are all filled with electrons, whereas Cu^2+^ ions have [Ar] 3d^9^ electron structures, which cause 2p→3d transitions with the result that satellite peaks are observed in XPS measurement [31,32,33,34]. As seen in Figure 1c,d, two satellite peaks were identified at 940 eV and 943 eV, except for the main peak at 933 eV in the Cu 2p_3/2_ core level. Therefore, the Cu 2p_3/2_ XPS spectra can be separated into Cu^+^, Cu^2+^, Cu(OH)_2_, and two satellite peaks (Sat1 and Sat2), and the ratio of Cu^+^ to Cu^2+^ can be expressed as Equations (4) and (5), respectively [19,35,36].

(4) $${\mathrm{Cu}}^{+}\left(\%\right)=\frac{{\mathrm{Cu}}^{+}}{{\mathrm{Cu}}^{+}+{\mathrm{Cu}}^{2+}+\mathrm{Cu}{\left(\mathrm{OH}\right)}_{2}+\mathrm{Sat}1+\mathrm{Sat}2}*100$$

(5) $${\mathrm{Cu}}^{2+}\left(\%\right)=\frac{{\mathrm{Cu}}^{2+}+\mathrm{Cu}{\left(\mathrm{OH}\right)}_{2}+\mathrm{Sat}1+\mathrm{Sat}2}{{\mathrm{Cu}}^{+}+{\mathrm{Cu}}^{2+}+\mathrm{Cu}{\left(\mathrm{OH}\right)}_{2}+\mathrm{Sat}1+\mathrm{Sat}2}*100$$

In the case of CuCo_2_O_4_-200 and CuCo_2_O_4_-300, the Cu^2+^ ion ratios were 81.3% and 81.4%, respectively. Co^3+^ and Co^2+^ ions also generated satellite peaks owing to the 2p→3d transition. Therefore, the Co 2p_3/2_ XPS spectra in Figure 1e,f can be separated into Co^3+^, Co^2+^ and two satellite peaks (Sat 1, Sat 2); the ratio for each ion can be expressed by Equations (6) and (7), respectively [37].

(6) $${\mathrm{Co}}^{3+}\left(\%\right)=\frac{{\mathrm{Co}}^{3+}+\mathrm{Sat}1}{{\mathrm{Co}}^{2+}+{\mathrm{Co}}^{3+}+\mathrm{Sat}1+\mathrm{Sat}2}*100$$

(7) $${\mathrm{Co}}^{2+}\left(\%\right)=\frac{{\mathrm{Co}}^{2+}+\mathrm{Sat}2}{{\mathrm{Co}}^{2+}+{\mathrm{Co}}^{3+}+\mathrm{Sat}1+\mathrm{Sat}2}*100$$

In the case of the CuCo_2_O_4_-200 and CuCo_2_O_4_-300, the Co^3+^ ion ratios were 70.9% and 72.3%, respectively. In *p*-type metal oxides, oxygen vacancies reduce the number of hole carriers, which has a significant influence on the electrical properties of the oxides [12,38]. The XPS measurement indicates that the solution-processed CuCo_2_O_4_ has no significant change in Cu and Co metal ion composition according to the annealing temperature change; nevertheless, there is a significant change in the oxygen defect. The O 1s peak measurements show that more oxygen defect sites were generated by low-temperature annealing in CuCo_2_O_4_-200. Therefore, we can expect that the hole concentration in CuCo_2_O_4_-200 is less than that in CuCo_2_O_4_-300. These results show the same tendency as the Hall measurement results, in which CuCo_2_O_4_-200 exhibited a lower hole concentration.

**Figure 2:**
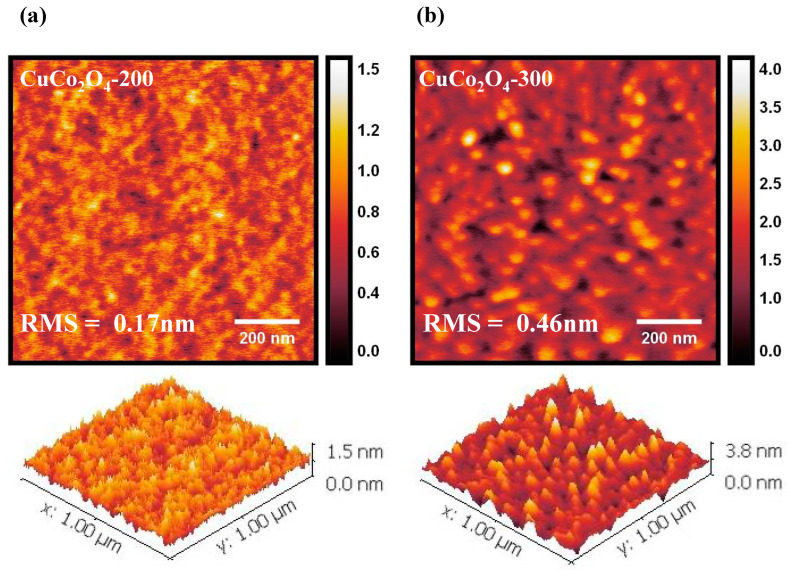
AFM topography image of (**a**) CuCo_2_O_4_-200 thin film and (**b**) CuCo_2_O_4_-300 thin film.

Figure 2 shows an AFM topography image of the CuCo_2_O_4_ thin films with different annealing temperatures measured in the non-contact mode. As shown in Figure 2a, the CuCo_2_O_4_-200 thin film has low crystallinity, whereas the CuCo_2_O_4_-300 thin film in Figure 2b shows that both the size and number of particles increased. Root mean square (RMS) value of CuCo_2_O_4_-200 and -300 thin films were measured to be 0.17 nm and 0.46 nm, respectively. The uniformity of the film formation seems to be the reason why the CuCo_2_O_4_-200 thin film had a higher hole mobility than CuCo_2_O_4_-300 (Table 1). As the CuCo_2_O_4_ annealing temperature decreased, crystallinity also decreased. Therefore, the low crystallinity of CuCo_2_O_4_-200 is a major factor in increasing the number of oxygen defect sites.

**Figure 3:**
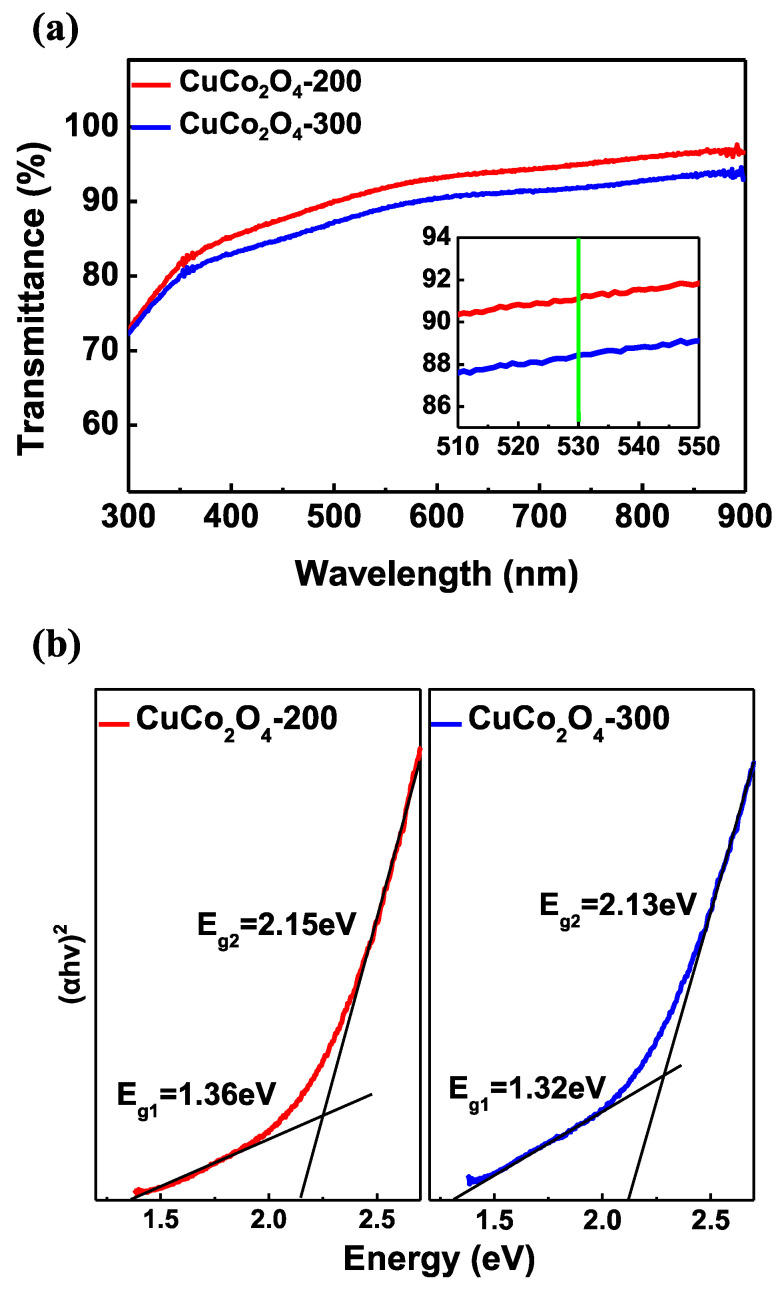
(**a**) Transmittance of CuCo_2_O_4_-200 and -300 thin film. (**b**) CuCo_2_O_4_-200 and -300 optical bandgap.

The optical characteristics of the CuCo_2_O_4_-200 and -300 films were measured by UV-visible spectroscopy. As shown in Figure 3a, the transmittance (%) of the CuCo_2_O_4_ films decreased as the annealing temperature was increased. Especially, the transmittance of CuCo_2_O_4_-200 and -300 samples was measured to be 91.1% and 88.4% respectively, in the 530 nm region, which is the QLED emission wavelength. The Tauc plots for the CuCo_2_O_4_ thin films annealed at different temperatures are shown in Figure 3b. The spinel oxides include divalent metal ions (M^2+^) and trivalent metal ions (M^3+^), and, thus, conduction bands formed by the 3d orbitals of M^2+^ and 3d orbitals of M^3+^ exist, respectively. Because both O 2p→M^2+^ 3d and O 2p→M^3+^ 3d transitions are generated, spinel oxides have two bandgap energies [39,40,41,42]. E_g1_ and E_g2_ in Figure 3b are the optical bandgaps generated by the O 2p→M^2+^ 3d and O 2p→M^3+^ transitions, respectively. The optical bandgap of CuCo_2_O_4_-200 with spinel structure was measured at 1.36 eV and 2.15 eV, and the bandgap of CuCo_2_O_4_-300 was measured at 1.32 eV and 2.13 eV. According to prior studies on spinel cobalt oxide, the crystallinity increases as the annealing temperature increases, whereas the bandgap and transmittance will decrease. The optical characterization of the CuCo_2_O_4_ covered in this study showed the same tendency as previous studies on spinel oxides [43]. Through UV-visible spectroscopy measurement results, owing to the low crystallinity of CuCo_2_O_4_-200, it was confirmed that the CuCo_2_O_4_-200 transmittance was higher than that of CuCo_2_O_4_-300 and, consequently, more suitable for QLED.

### 3.2. Hole Injection Ability of CuCo_2_O_4_ HIL

**Figure 4:**
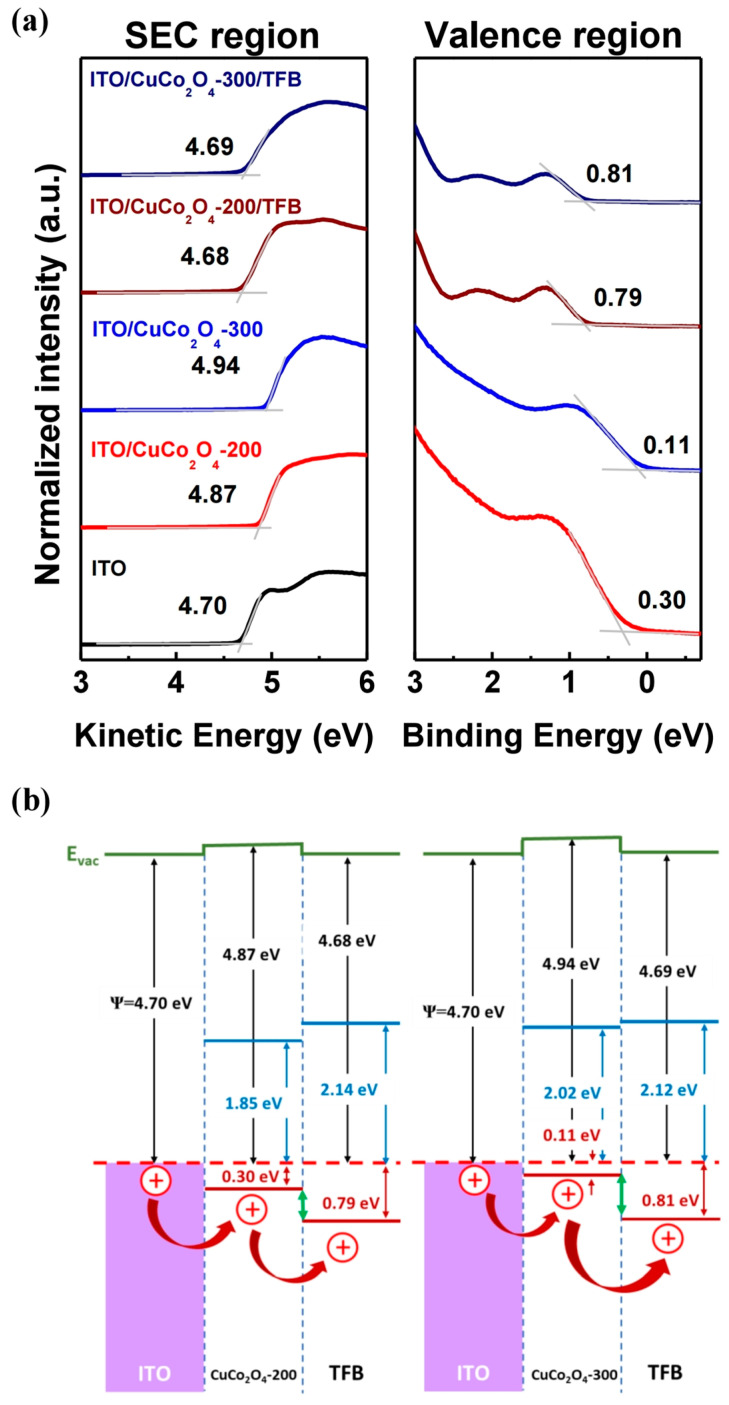
(**a**) UPS spectra of ITO, ITO/HILs and ITO/HILs/HTL measured SEC (left) and valence region (right). (**b**) Detailed interfacial energy-level alignment of CuCo_2_O_4_ with ITO and TFB.

To identify the hole injection mechanism of QLEDs through the electronic structure analysis of the CuCo_2_O_4_ HIL, UPS measurements of ITO, ITO/CuCo_2_O_4_-200, ITO/CuCo_2_O_4_-300, ITO/CuCo_2_O_4_-200/TFB, and ITO/CuCo_2_O_4_-300/TFB samples were performed. In Figure 4a, the graph on the left shows the work function measurement based on the kinetic energy of electrons in the secondary-electron cutoff region. In Figure 4a, the graph on the right shows the energy difference between the Fermi energy level (E_F_) and the VBM or highest occupied molecular orbital (HOMO) through the binding energy of electrons. The work functions of samples ITO/CuCo_2_O_4_-200, ITO/CuCo_2_O_4_-300, ITO/CuCo_2_O_4_-200/TFB and ITO/CuCo_2_O_4_-300/TFB were measured to be 4.87 eV, 4.94 eV, 4.68 eV, and 4.69 eV, respectively, then the VBM (or HOMO) were measured to be 0.30 eV, 0.11 eV, 0.79 eV, and 0.81 eV, respectively. The optical band gap of TFB was estimated using the Tauc plot (Figure S3). The work function difference and HOMO level difference between the TFB thin film formed on CuCo_2_O_4_-200 and formed on CuCo_2_O_4_-300 were 0.01 eV and 0.02 eV, respectively, and these were not significant. However, in the CuCo_2_O_4_ HILs, an energy level difference appeared when the annealing temperature changed, and the VBM in particular changed significantly. The CuCo_2_O_4_-300 HIL has few oxygen defect sites owing to its high crystallinity, which increases the hole concentration and correspondingly reduces the gap between the VBM and E_F_ levels. Contrastingly, the CuCo2O4-200 HIL has many oxygen defect sites, which decreases the hole concentration and correspondingly increases the gap between the VBM and E_F_ levels. As shown in Figure 4b, the difference between the CuCo_2_O_4_-300 VBM and TFB HOMO levels was 0.7 eV, whereas the difference between CuCo_2_O_4_-200 and TFB HOMO levels was 0.49 eV, which is relatively small. The UPS measurement results suggested that the CuCo_2_O_4_-200/TFB structure was more suitable for the device in terms of hole injection.

**Figure 5:**
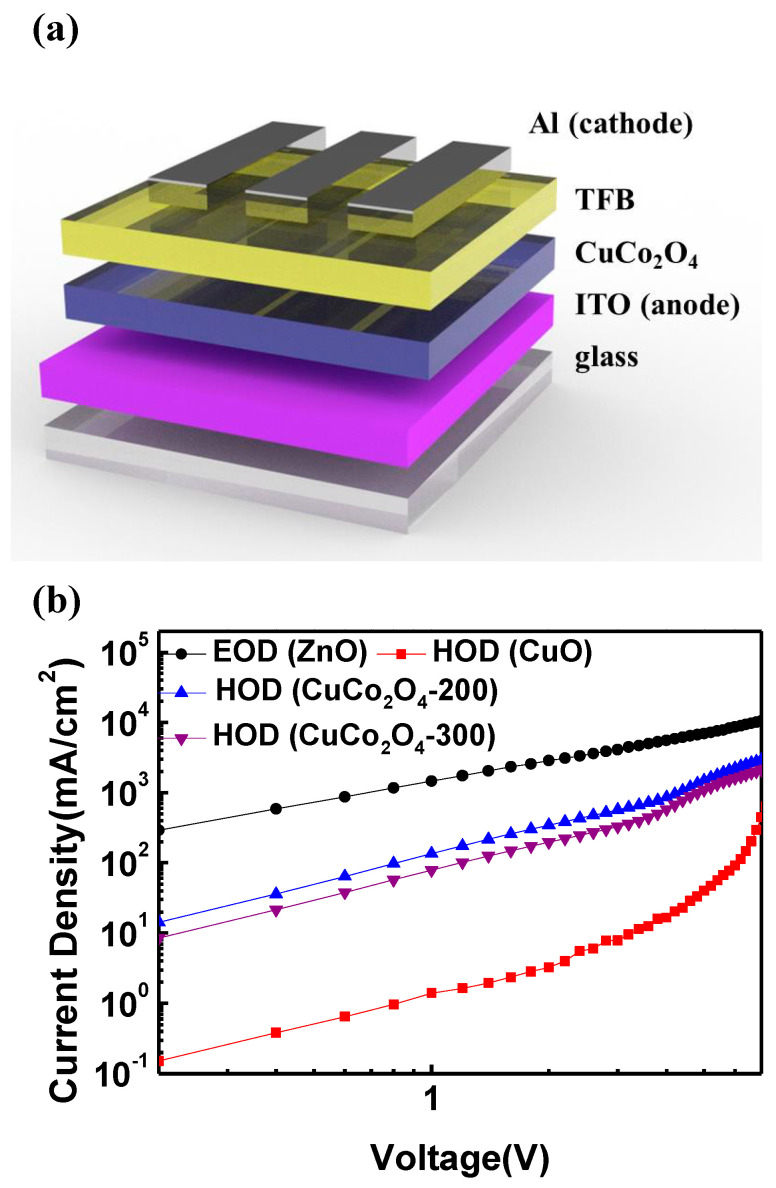
(**a**) Schematic structure of HOD with a CuCo_2_O_4_ HIL and (**b**) current density-voltage (J-V) characteristics of HODs and the EOD.

To confirm the degree of hole injection in actual devices according to the CuCo_2_O_4_ HILs annealing temperature, the J-V characteristics were measured by manufacturing an HOD of the ITO/CuCo_2_O_4_/TFB/Al structure, as displayed in Figure 5a. An EOD with an ITO/ZnO/Al structure was also fabricated to compare the injection and transport properties of the electrons and hole carriers. Figure 5b shows that more current flows in the EOD than in the HOD, which can be expected based on the characteristics of holes and electrons. Remarkably, the current density of the HODs using the ternary metal oxide CuCo_2_O_4_ HIL was nearly 100 times higher than that of the binary metal oxide CuO. In addition, as expected from the UPS measurement results, it can be confirmed that hole injection increases in the CuCo_2_O_4_-200 HOD, which exhibits suitable energy alignment with the TFB. Therefore, we expect that the CuCo_2_O_4_ HIL can improve the performance of QLEDs owing to its high hole injection ability, and in particular, that CuCo_2_O_4_-200 HIL QLEDs will have the highest performance.

### 3.3. CuCo_2_O_4_ HIL-Based QLED Structure and Performance

**Figure 6:**
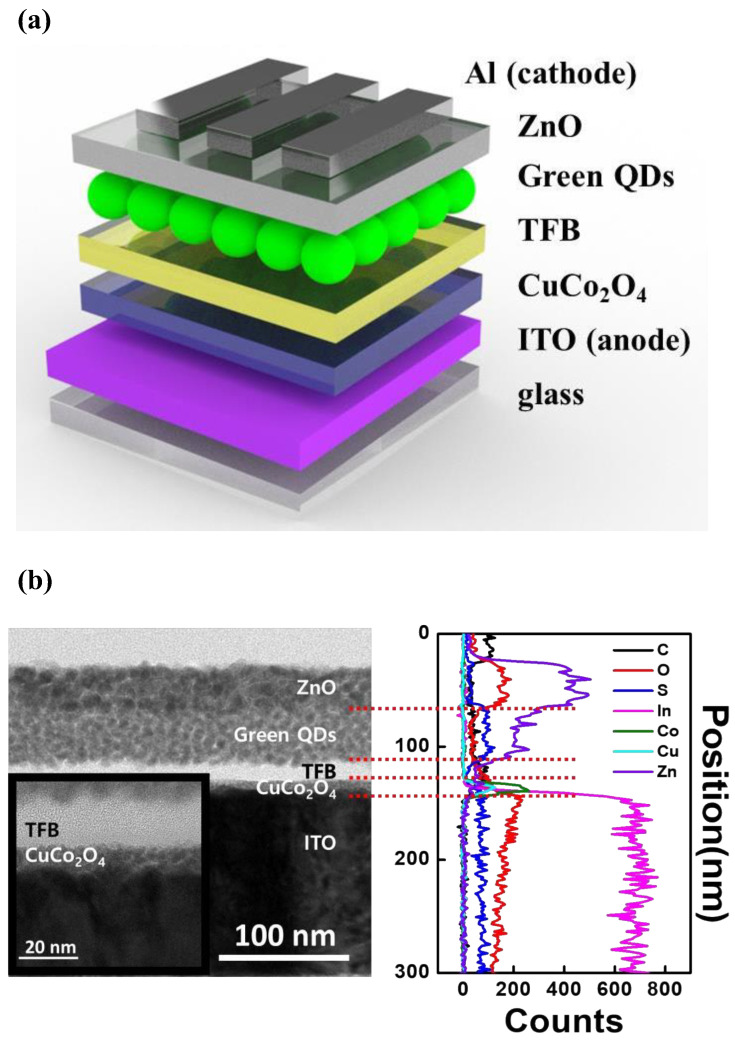
(**a**) Structure of QLEDs with CuCo_2_O_4_ HIL. (**b**) Cross-sectional HR-TEM image of the QLED with CuCo_2_O_4_ HIL and EDS line scan of the elemental distribution.

Figure 6a shows the structure of the QLED that was fabricated using the solution process. First, the CuCo_2_O_4_, TFB, QDs, and ZnO layers were prepared by spin-coating on a patterned glass/ITO substrate. Then, the 130 nm Al cathode was thermally evaporated using a metal shadow mask. Figure 6b shows a cross-sectional HR-TEM image of the QLED fabricated using the solution process. The HR-TEM image shows that CuCo_2_O_4_/TFB/QDs/ZnO thin films were formed with uniform thickness. The thicknesses of the CuCo_2_O_4_-200, TFB, QD, and ZnO layers in the TEM images were 8, 13, 40, and 35 nm, respectively. EDS line scanning (Figure 6c) displayed the overall elemental distribution, confirming that each solution-processed thin film was well separated. The EDS mapping images of C, O, S, In, Co, Cu, and Zn are shown in Figure S4. In the EDS data, copper (Cu) and cobalt (Co) peaks are co-located, indicating that Cu and Co do not separate and form a monolayer. In particular, the atomic ratio of the CuCo_2_O_4_ layer analyzed by EDS was Cu:Co:O = 0.9:2:4.1 (Figure S5). This atomic ratio provides evidence of the formation of a stable spinel (AB_2_O_4_) structure.

**Figure 7:**
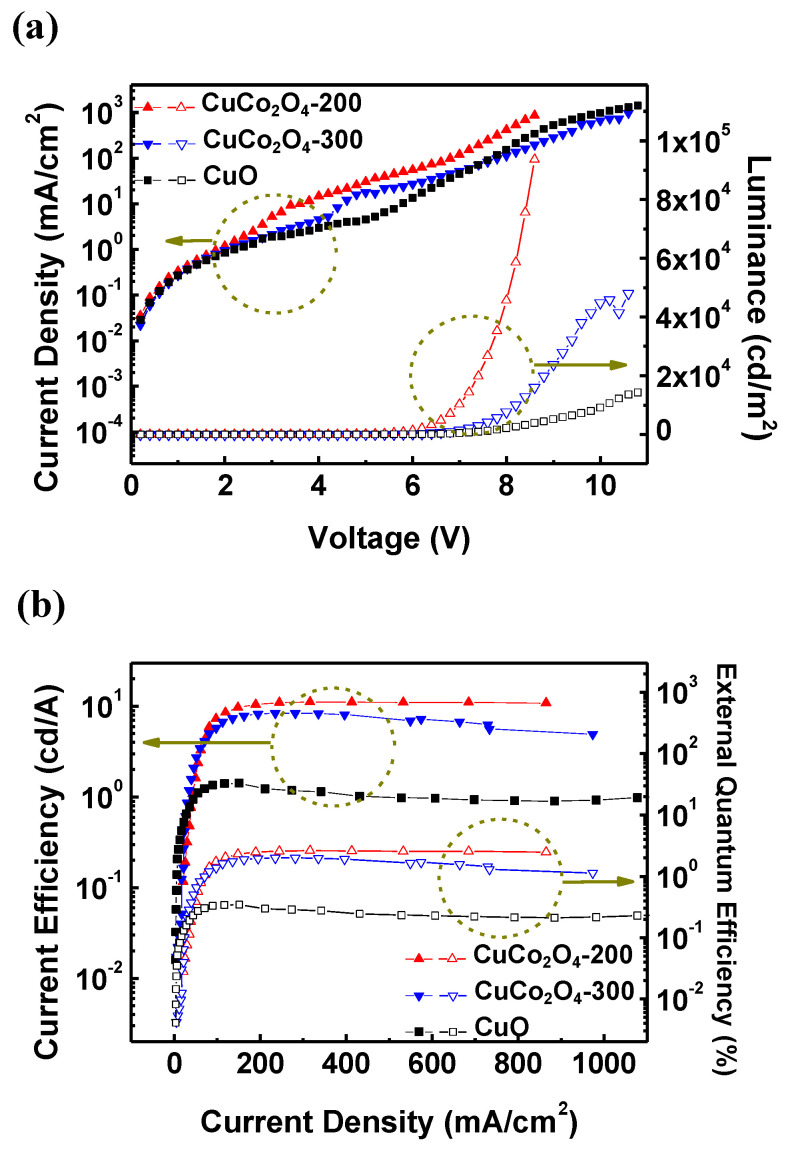
Performance of the QLEDs with CuCo_2_O_4_ HILs. (**a**) Current density-voltage-luminance (J-V-L) curves. (**b**) Current efficiency-current density-external quantum efficiency (CE-J-EQE) curves.

Figure 7 shows the light-emitting performance of the CuCo_2_O_4_ and CuO-HIL-based QLEDs. Figure 7a shows the current density-voltage-luminance (J-V-L) characteristics of QLEDs, and all CuCo_2_O_4_ HIL-based QLEDs show a higher luminance than the CuO HIL-based QLED. The performance improvement of the QLEDs is owing to the excellent hole injection characteristics of CuCo_2_O_4_ and shows the same tendency as the J-V characteristics of the HOD in Figure 5b. Furthermore, as expected, the energy barrier between CuCo_2_O_4_-200 VBM and TFB HOMO was smaller than that of CuCo_2_O_4_-300, which was advantageous in terms of hole injection, and the CuCo_2_O_4_-200 HIL QLED had the highest performance. The device with the CuCo_2_O_4_-200 HIL showed the highest luminance of 93,607 cd/m^2^. The current efficiency of the QLEDs with CuCo_2_O_4_-200 HIL was measured as 11.11 cd/A, which was considerably enhanced compared to that of the device with CuO HIL. In addition, for the CuCo_2_O_4_ HIL QLED, the full width at half maximum (FWHM) of the emission wavelength at the maximum luminance was also reduced to 24 nm (Figure S6). Additionally, the Commission internationale de l’eclairage 1931 (CIE 1931) (x, y) coordinates of the CuCo_2_O_4_-200 HIL QLED were measured as (0.214, 0.751), this shows that the CuCo_2_O_4_-200 HIL QLED emits more monochromatic green light than the CuCo_2_O_4_-300 and CuO HIL QLEDs. The measured performance data of the devices are summarized in Table 2. As shown in Figure 7, the current density at low voltage of the CuCo_2_O_4_ HIL QLEDs was higher than that of the CuO HIL QLED. However, the current density of the CuO HIL QLED rapidly increased after turning on the device, whereas the CuCo_2_O_4_-based device showed a gradual increase rate of the current density due to a decrease in the leakage current of the device. Therefore, the current efficiency was higher for QLEDs with CuCo_2_O_4_ HIL than CuO HIL and a narrow FWHM could be realized, allowing higher color purity of the device. This is due to the enhanced charge balance of the device with better hole injection. This study demonstrates that efficient hole injection can be achieved using a solution-processed spinel CuCo_2_O_4_ HIL in QLEDs.

## 4. Conclusions

In this study, we fabricated CuCo_2_O_4_ HIL-based QLEDs to increase hole injection and improve luminous efficiency. The electrical properties of CuCo_2_O_4_ comprising Cu^2+^ and Co^3+^ cations were measured using Hall measurements, and it was confirmed that they have higher conductivity than common *p*-type binary oxides. In particular, the difference in the hole injection ability of CuCo_2_O_4_ according to the annealing temperature was identified using XPS, UPS, and the fabricated HOD. It was confirmed that the VBM of CuCo_2_O_4_ shifted away from the Fermi energy level (E_F_) as the annealing temperature decreased, resulting in enhanced hole injection through better energy-level alignment. The CuCo_2_O_4_ HODs had a significantly higher current density than the CuO HOD, and in particular, the hole injection ability of the CuCo_2_O_4_-200 HOD was superior to that of the CuCo_2_O_4_-300 HOD, as expected from the perspective of energy alignment. Maximum luminance and maximum current efficiency of the CuCo_2_O_4_-200 HIL QLED were 93,607 cd/m^2^ and 11.14 cd/A, respectively, resulting in a 656% improvement in luminous performance of QLEDs compared to the CuO HIL QLED owing to increased hole injection. This study demonstrates that efficient hole injection can be achieved using a solution-processed spinel CuCo_2_O_4_ HIL in QLEDs.

## Figures and Tables

**Table 1:** Hall measurement results of the CuCo_2_O_4_ at different annealing temperature.

Sample	Resistivity (Ω·cm)	Mobility (cm^2^/V·s)	Hole concentration (cm^−3^)
CuCo_2_O_4_-200	2.295	0.96	+2.834 × 10^18^
CuCo_2_O_4_-300	0.3917	0.72	+2.214 × 10^19^
CuO	N/A	N/A	N/A

**Table 2:** Summarized device performances of QLEDs at different CuCo_2_O_4_ annealing temperature.

Sample	L_max_ (cd/m^2^)	CE_max_ (cd/A)	EQE_max_ (%)	FWHM (nm)	CIE 1931 (x, y)
CuCo_2_O_4_-200	93,607	11.14	2.62	24	(0.214, 0.751)
CuCo_2_O_4_-300	47,935	8.42	2.00	24	(0.219, 0.747)
CuO	14,268	1.42	0.34	26	(0.249, 0.725)

## Data Availability

Data will be made available from the corresponding authors on reasonable request.

## Supplementary Materials

The following supporting information can be downloaded at: https://www.mdpi.com/article/10.3390/ma16030972/s1, Figure S1. Crystallographic structure of AB2O4 spinel. Figure S2. (a) The optical band gap of CdSe/ZnS green QDs. (b) The Photoluminescence spectra of CdSe/ZnS green QDs. Figure S3. The optical band gap of TFB was estimated using the Tauc plot. Figure S4. EDS mapping image of various elements (C, O, S, In, Co, Cu, Zn and Se) in the CuCo2O4 HIL based-QLED. Figure S5. EDS line scan data of atomic percent. Figure S6. Normalized EL spectra of QLED at the maximum luminance.
