# Supplementary material for: Enhancing the Performance of Quantum Dot Light-Emitting Diodes Using Solution-Processable Highly Conductive Spinel Structure CuCo2O4 Hole Injection Layer
Source: Materials (Basel). 2023 Jan 20;16(3):972. doi: 10.3390/ma16030972 (PMC9919813; doi:10.3390/ma16030972)
Supplement: Supplementary file 1 [file materials-16-00972-s001.zip › materials-2176761-SI.pdf]

Supporting Information

Enhancing the Performance of Quantum Dot Light-Emitting Diodes Using Solution-Processable Highly Conductive Spinel Structure $CuCo_2O_4$ Hole Injection Layer

Min Ho Park [1,2], Min Gye Kim [1,2], Jin Hyun Ma [1,2], Jun Hyung Jeong [1,2], Hyoun Ji Ha [1,2], Wonsik Kim [3], Soohyung Park [3,4] and Seong Jun Kang [1,2,*]

[1] Department of Advanced Materials Engineering for Information and Electronics, Kyung Hee University, Yongin 17104, Republic of Korea
[2] Integrated Education Program for Frontier Materials (BK21 Four), Kyung Hee University, Yongin 17104, Republic of Korea
[3] Advanced Analysis Center, Korea Institute of Science and Technology, 5 Hwarang-ro 14-gil, Seongbuk-gu, Seoul 02792, Republic of Korea
[4] Division of Nano & Information Technology, KIST School, University of Science and Technology (UST), Seoul 02792, Republic of Korea
* Correspondence: junkang@khu.ac.kr

Index

Figure S1. Crystallographic structure of AB_2O_4 spinel

Figure S2. (a) The optical band gap of CdSe/ZnS green QDs. (b) The Photoluminescence spectra of CdSe/ZnS green QDs.

Figure S3. The optical band gap of TFB was estimated using the Tauc plot

Figure S4. EDS mapping image of various elements (C, O, S, In, Co, Cu, Zn and Se) in the $CuCo_2O_4$ HIL based-QLED

Figure S5. EDS line scan data of atomic percent

Figure S6. Normalized EL spectra of QLED at the maximum luminance

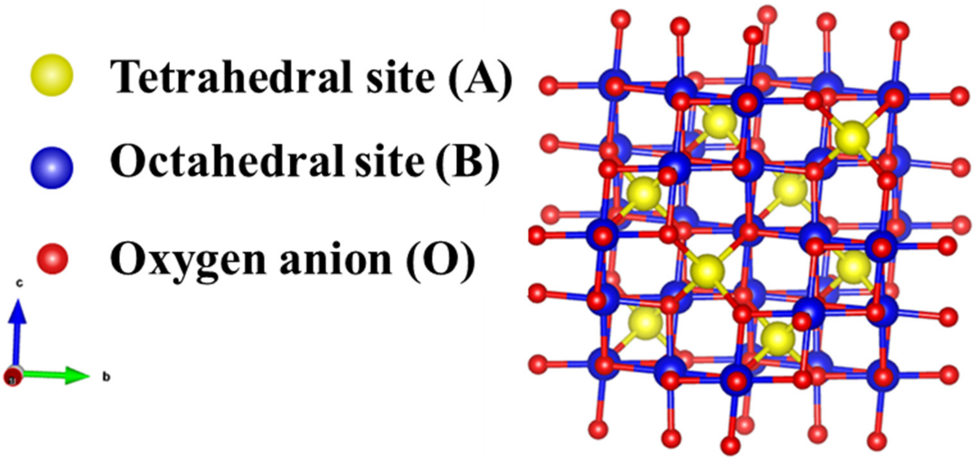

Figure S1. Crystallographic structure of AB_2O_4 spinel

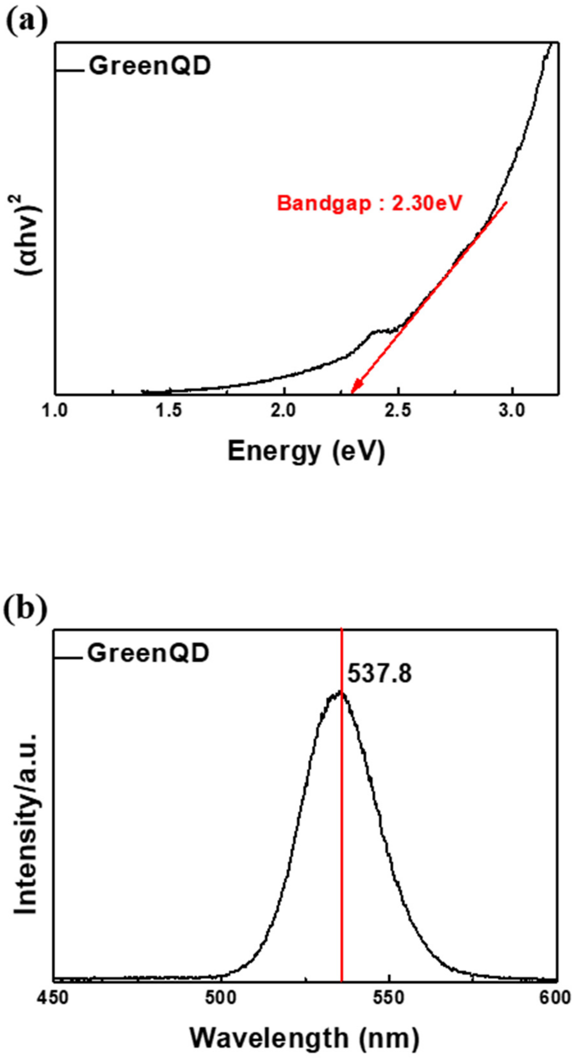

Figure S2. (a) The optical band gap of CdSe/ZnS green QDs. (b) The Photoluminescence spectra of CdSe/ZnS green QDs

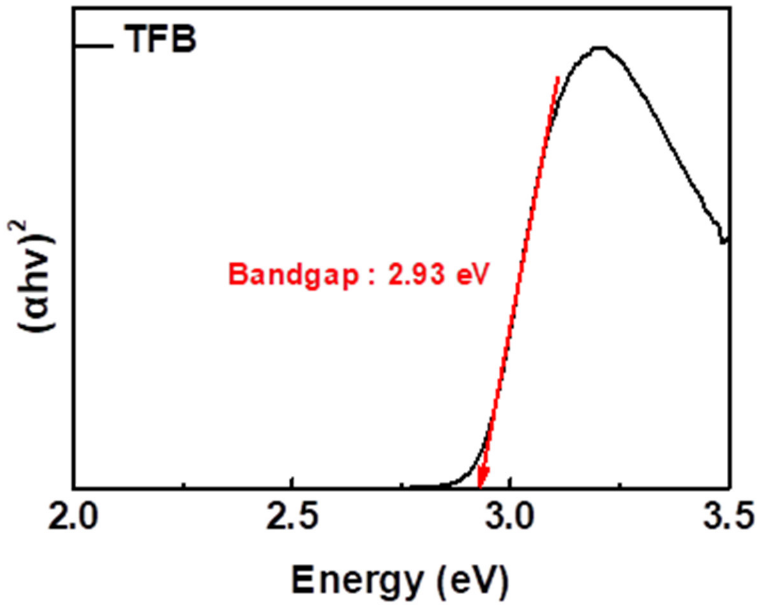

Figure S3. The optical band gap of TFB was estimated using the Tauc plot

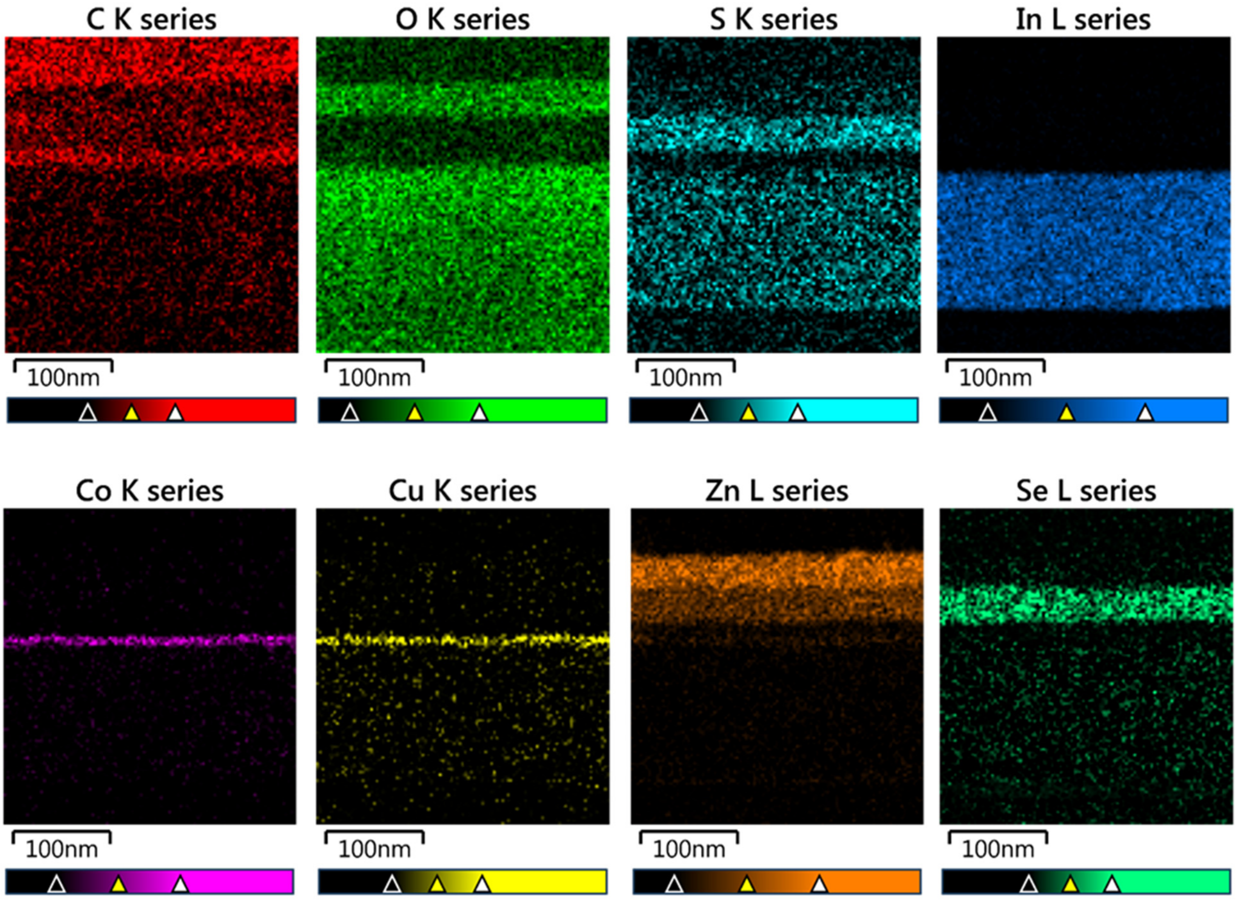

Figure S4. EDS mapping image of various elements (C, O, S, In, Co, Cu, Zn and Se) in the $CuCo_2O_4$ HIL based-QLED

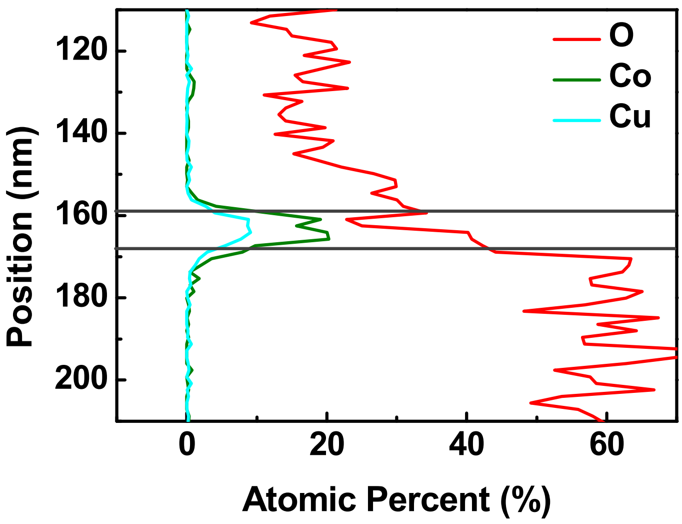

Figure S5. EDS line scan data of atomic percent

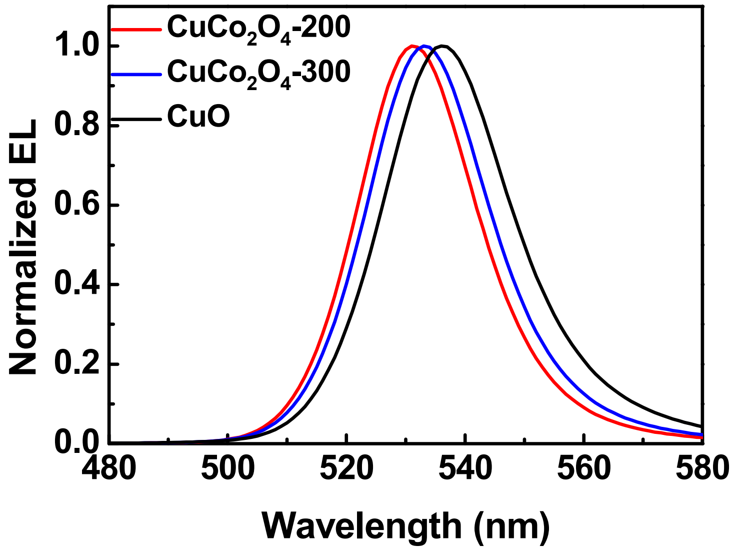

Figure S6. Normalized EL spectra of QLED at the maximum luminance
